# Bone Conduction Capacity of Highly Porous 3D-Printed Titanium Scaffolds Based on Different Pore Designs

**DOI:** 10.3390/ma14143892

**Published:** 2021-07-12

**Authors:** Ho-Kyung Lim, Miyoung Ryu, Su-Heon Woo, In-Seok Song, Young-Jun Choi, Ui-Lyong Lee

**Affiliations:** 1 Department of Oral and Maxillofacial Surgery, Korea University Guro Hospital, Seoul 08308, Korea; ungassi@naver.com; 2 CUSMEDI Inc., Sungnam 13217, Korea; miyung0829@gmail.com; 3 R&D Center, Medyssey Co., Ltd., Jechon 27159, Korea; su-heon.woo@medyssey.com; 4 Department of Oral and Maxillofacial Surgery, Korea University Anam Hospital, Seoul 02841, Korea; densis@korea.ac.kr; 5 Department of Oral & Maxillofacial Surgery, Chungang University Hospital, Seoul 03080, Korea; oms@hanmail.net

**Keywords:** titanium scaffolds, 3D printing, pore design, selective laser melting

## Abstract

In porous titanium scaffolds manufactured via 3D printing, the differences in bone formation according to pore design and implantation period were studied. Titanium scaffolds with three types of different pore structures (Octadense, Gyroid, and Dode) were fabricated via 3D printing using the selective laser melting method. Mechanical properties of scaffolds were investigated. Prepared specimens were inserted into both femurs of nine rabbits and their clinical characteristics were observed. Three animals were sacrificed at the 2nd, 4th, and 6th weeks, and the differences in bone formation were radiologically and histologically analyzed. The percentage of new bone and surface density in the pore structure were observed to be approximately 25% and 8 mm^2^/mm^3^, respectively. There was no difference in the amount of newly formed bone according to the pore design at 2, 4, and 6 weeks. In addition, no differences in the amount of newly formed bone were observed with increasing time within the same pore design for all three designs. During the 6-week observation period, the proportion of new bones in the 3D-printed titanium scaffold was approximately 25%. Differences in bone formation according to the pore design or implantation period were not observed.

## 1. Introduction

Titanium has been successfully applied to humans in various fields currently. Titanium is widely used in various implantable medical devices such as dental implants, bone fixators, artificial joints, vascular stents, and shielding membranes [1]. Because titanium is not resorbed like polymers or some ceramics, the application of titanium as a scaffold for bone growth is still not extensive compared to that of ceramic materials [2]. However, titanium, which has higher tensile and compressive strength than ceramic and similar elastic modulus to human bone, is recognized for its high clinical value as bone grafting material [3].

The porous structures of titanium are considered to be effective for eliminating elastic modulus mismatches with human bone [4]. Additionally, the porous structure provides space for bone formation and regrowth [5]. However, accurately controlling the pore size, porosity, shape, and connectivity of the porous structures of titanium scaffolds manufactured by conventional casting or milling methods is difficult [6]. To overcome this problem, various manufacturing methods such as sintering of metal fibers, plasma spraying, anodic dissolution, or powder metallurgy have been introduced; however, constructing a homogenous porous structure is still difficult [7]. In addition, fabricating a small-sized scaffold with a microstructure using a conventional manufacturing method was impossible [8]. However, 3D printing, which has been actively researched and used in recent years, has made it possible to overcome such manufacturing imperfections. Based on a fast, uniform, and simple process through 3D printing, producing a metallic scaffold with a homogeneous porous structure has become possible [9].

It has been reported that the porosity of the ideal scaffold for bone regeneration is more than 66% [10]. Moreover, the pore shape of a scaffold affects bone formation through cell growth. Bidan et al. demonstrated that the optimization of the pore shape can increase the growth rate of the bone tissue in a porous scaffold, and the cells grow faster in square pores [11]. The results of Urda et al. indicate that the straight and low convexities in the pore structure are the least favorable for cell growth [12]. Van Bael et al. studied the pore shape and curvature, and the results showed that an obtuse angle between the pores was more prone to cause blockage of cell growth than an acute angle [13]. However, these studies were conducted at an in vitro level, and the results of in vivo experiments on the effect of pore structure and bone formation were inconsistent [14,15].

In this study, under the premise that the specimens have the same porosity, titanium alloy scaffolds with microstructures of three different shaped pore designs were fabricated using 3D printing. In addition, we analyzed whether there was a difference in bone formation according to pore design or implantation period in titanium scaffolds in vivo using rabbit animal model.

## 2. Materials and Methods

### 2.1. Selection of the Pore Design

Among the various mesh designs provided by the Magics 21.0 program (Materialise NV, Leuven, Belgium), 5 designs with a pore size of 1 mm were selected—Diamond, Cubic, Dode, Octadens, Gyroid. Before specimen production for the experiment, non-destructive analysis was performed using micro-computed tomography (μCT) (Quantum FX micro-CT, Perkin Elmer Inc., Hopkinton, MA, USA) to analyze the structure of the lattice shape. After obtaining μCT images of each pore design sample, 3D images were rendered with NRecon program (Bruker-CT, Kontich, Belgium). BoneJ tap of ImageJ software (NIH and LOCI, University of Wisconsin, Madison, WI, USA) for bone micro-architecture analysis was used for stereology analysis of the output design. After cropping a certain area (TV, 125 mm^3^) from the entire μCT image, scaffold volume ratio was calculated on the program. It was observed that the Dode (0.708%), Octadens (0.828%), and Gyroid (0.775%) designs had relatively higher densities than the Diamond (0.455%) and Cubic (0.447%) designs. Therefore, it was decided to manufacture Dode, Octadens, and Gyroid designs for experimental specimens.

### 2.2. Manufacture of 3D-Printed Titanium Specimens

**Figure 1:**
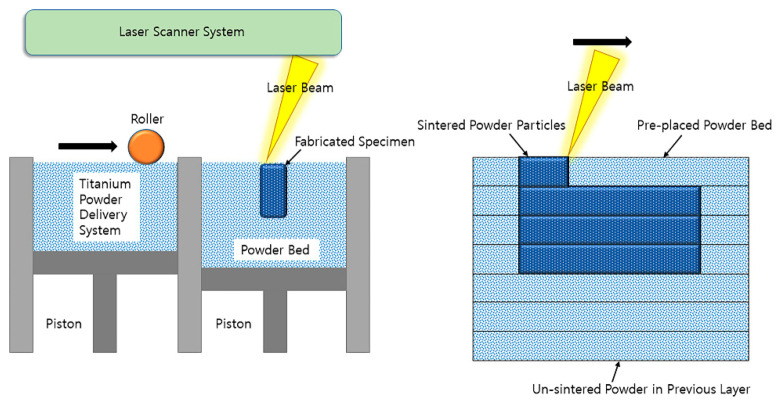
Selective laser melting type 3D-printing process of highly porous titanium specimens.

**Figure 2:**
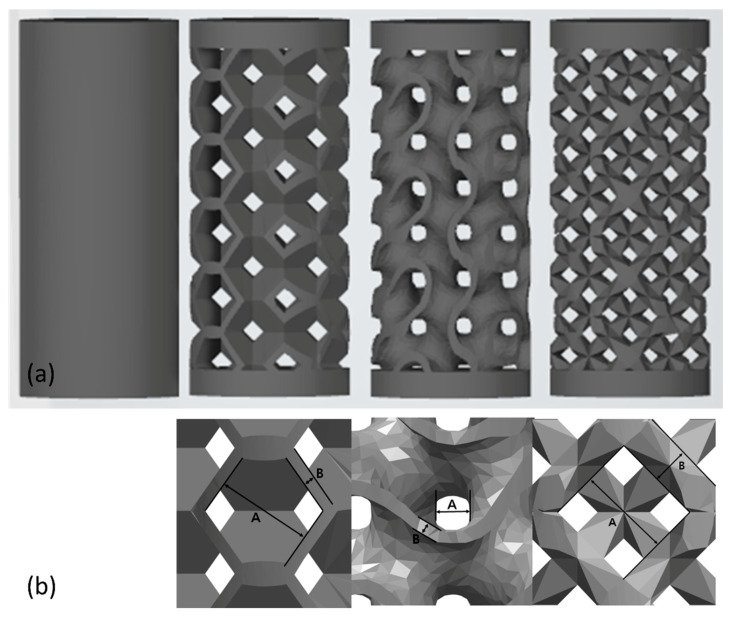
Stereolithographic image of four different pore designs. (**a**) Entire shape (Solid (sham), Octadens, Gyroid, and Dode). (**b**) Image of the unit cell (Octadens, Gyroid, and Dode) (A: pore size, B: strut thickness).

A 3D printing method was adopted with the selective laser melting (SLM) (Cusmedi^©^, Sungnam, Korea) fabrication method. All manufacturing processes were managed in accordance with the Good Manufacturing Practice standards of the Korean Ministry of Food and Drug Safety. The raw material for printing was Ti-6Al-4 V-ELI powder (Arcam A2, Arcam, Moindal, Sweden) suitable for the ASTM F3001-14 (Standard specification for additive manufacturing Ti-6Al-4 V-ELI with powder bed fusion). The composition of the powder was mainly titanium, which contained trace elements such as 5.94% aluminum, 4.14% vanadium, 0.008% carbon, 0.049% iron, 0.010% yttrium, 0.10% oxygen, 0.010% nitrogen, and less than 0.002% zinc and hydrogen (in wt.%). The manufacturing process based on the SLM system was as follows: Thin layers of atomized fine titanium powder were evenly distributed onto a substrate plate via selective laser melting using a coating mechanism. This process occurred inside a chamber with a strictly controlled atmosphere of inert nitrogen and argon. Once each layer had been stacked, each 2D slice of the part geometry was fused by melting the powder selectively. The laser energy was sufficiently intense to permit complete welding of the particles to form a solid and hard metal. This process was accomplished by a high-powered laser beam, which is usually composed an ytterbium fiber. The process was repeated layer-by-layer until the manufacturing process was complete (Figure 1). The standard of the manufactured product was the ASTM F136 (Grade 23, Standard specification for Ti-6Al-4 V-ELI alloy). Through the manufacturing process, cylinder-shaped specimens with four different pore designs were prepared: Solid (sham, no pore), Octadense, Gyroid, and Dode. All specimens have isotropy in which the same shape is repeated in the X, Y, and Z planes, and these structures also have similar mechanical strength along the X, Y, and Z axes. The specimens were 7 mm in height and 3 mm in diameter (Figure 2). Except for the solid, the specimens of the other designs were manufactured with the same porosity of 75%. The pore size of Octedens, Gyroid, and Dode design were 1.07, 0.30, and 0.76 mm, respectively. The strut thickness of Octedens, Gyroid, and Dode design were 0.12, 0.14, and 0.30 mm, respectively. The strut spacing of all specimens except solid was 0.8 mm, and the printing error was set within 0.05 mm. Surface roughness was less than 15um, and Vickers hardness was more than 310 VHN.

### 2.3. Animal Experiments

These animal experiments were approved by the Animal Laboratory Ethics Committee based on the law on laboratory animals (Approval No: CRONEX-IACUC 201908-001).

**Figure 3:**
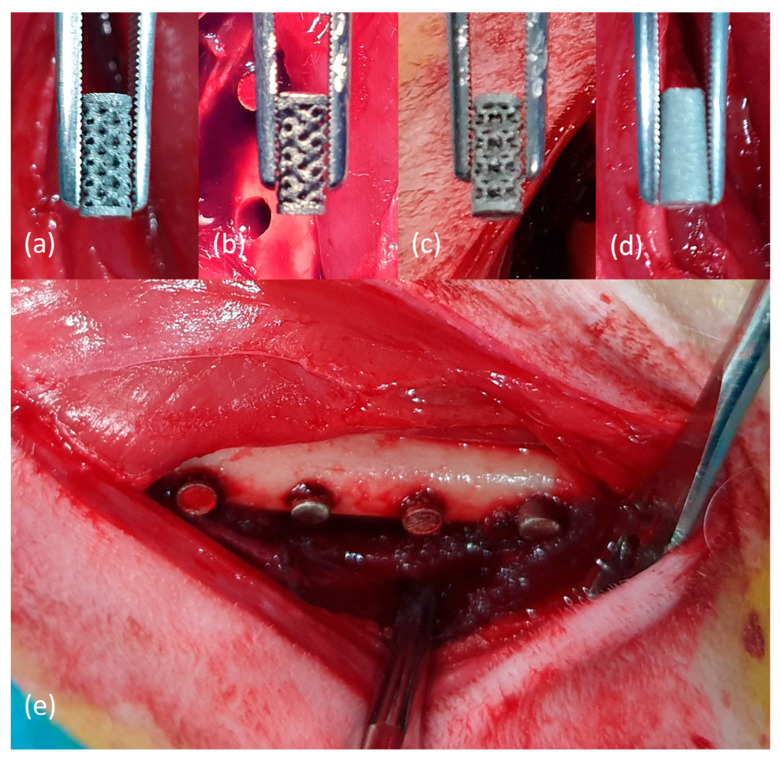
(**a**–**d**) Cylinder-shaped highly porous 3D-printed titanium specimens with four different pore designs (Octadens, Gyroid, Dode, and Solid (sham)). (**e**) Application and insertion of 3D-printed titanium specimens onto the rabbit medial femur.

Nine twenty-week-old New Zealand white rabbits (weight: 3.6–3.8 kg) were used herein. After one week of acclimatization, general anesthesia using a combination of zolazepam and tiletamine (Zoletil^®^, 50 mg/kg, Virbac Korea, Seoul, Korea) and xylazine HCl (Rompun^®^, 10 mg/kg, Bayer Korea, Seoul, Korea) was administered intramuscularly. On one side of the shaved femur, a disinfection agent with betadine was applied. Subsequently, lidocaine with 1:100,000 epinephrine was administered subcutaneously for hemostatic purpose, and an incision of approximately 4 cm was made longitudinally on the inner thigh. Complete bony exposure of the medial aspect of the femur was performed through careful dissection between the rectus femoris and vastus medialis muscles. Four cuts at a width of 3 mm were made using drills for the installation of specimens. The gap between the cuts was 7 mm. The prepared specimens were individually inserted into each cut (Figure 3). To minimize the effect of the installation location, each different pore-designed specimen was inserted into a random location. After inserting the specimens, layer-to-layer suturing was performed using 4-0 Vicryl^®^ and 5-0 Ethylon^®^ (Johnson & Johnson, New Brunswick, NJ, USA). The same surgical procedure was performed on the opposite leg.

For pain relief and prevention of infection, prednisolone (0.5 IM, IM, Yuhan, Seoul, Korea) and cephalexin (5 mg/kg, IM, Yuhan, Seoul, Korea) were postoperatively injected for two days. The animals were monitored weekly for infection, inflammation, wound dehiscence, delayed healing, and general health until they were euthanized. Out of a total of nine rabbits, three were euthanized at the 2nd, 4th, and 6th weeks. After being anesthetized using a zolazepam, tiletamine, and xylazine HCl, the animals were euthanized by administering potassium chloride to the marginal ear vein. After euthanasia process, the surgical site of the femur was dissected supra-periosteally, and the long bone and adjacent soft tissue of the femur were harvested.

### 2.4. Micro-CT Imaging and Volumetric Analysis

The extracted femurs were moved and fixed in a μCT machine (SkyScan1174^®^, Ver. 1.7; Bruker CT, Kontich, Belgium). Subsequently, μCT imaging was taken. The imaging settings were as follows—tube voltage of 130 kVp, tube current of 60 μA, aluminum filter of 1 mm, exposure time of 500 ms, and rotation angle of 0.3°. The pixel size of image was 13.86 μm. The number of pixels of each image cut was 2240 × 2240. Under these circumstances, a total of 800 high-resolution image cuts were obtained. NRecon (Ver 1.7.0.5, Bruker-CT, Kontich, Belgium) was used for reconfiguration of cross-sectional image cuts, and Dataviewer (Ver. 1.5.1.3, Bruker-CT, Kontich, Belgium) were used for the construction of 3D images. The volume of newly formed bone inside the pore of the specimen was calculated using the difference at the grayscale level. The formula for calculating the new bone volume is as follows:

(Percentage of) New bone volume (%) = (Percentage of) Total bone volume − (Percentage of) Grafted specimen volume

The surface density of newly formed bone inside the pores of the specimen was calculated in the same manner. The formula for calculating the surface density of a new bone is as follows:

Surface density of new bone (mm^2^/mm^3^) = segmented bone surface/total new bone volume of the region of interest.

### 2.5. Histological Findings

Tissue slides were prepared using a non-decalcified sample preparation method. The harvested long bone and adjacent soft tissue of the femur were fixed with 10% formalin solution. After soaking of the specimens in formalin solution for one week, they were rinsed meticulously with massive water for 12 h. The specimens were dehydrated using increasing concentrations of 70%, 90%, and 100% ethanol. Then, the tissue samples were infiltrated with resin solution (Technovit 7200 resin, Heraeus KULZER, Hanau, Germany) with increasing resin ratios. After penetration by vacuuming the resin solution for two days, curing of the resin was performed using a UV Embedding system (KULZER EXAKT 520^®^, Heraeus KULZER, Hanau, Germany), and cut of the center of the resin block was done using a diamond cutter to produce a cross-section of the embedded tissue until 40 μm thickness. Sectioned thin tissue was stained using hematoxylin and eosin (H&E), and tissue slides were produced. The images of tissue slides were scanned using an optical microscope (OLYMPUS BX^®^, Olympus Optical CO. Tokyo, Japan) equipped with a complementary metal–oxide–semiconductor sensored camera. Subsequently, the histological images were observed.

### 2.6. Statistical Interpretation

The Kruskal–Wallis test was used to analyze the differences in the percent volume and the surface density of new bone among the three different pored groups simultaneously. Moreover, the Kruskal–Wallis test was used to compare the percent volume and the surface density of new bone at 2, 4, and 6 weeks within the group. Statistical analysis was performed using SPSS 22 program (IBM SPSS Inc., Chicago, IL, USA). Statistical significance was designated at *p* < 0.05.

## 3. Results

### 3.1. Clinical Findings in the Animal Experiment

During the observational period, the animals did not show any life-threatening clinical signs. In addition, abnormal symptoms such as wound dehiscence, infection, or inflammation were not observed at the operated sites. Significant gait impairment was not observed during the observational period.

### 3.2. Radiological Findings

**Figure 4:**
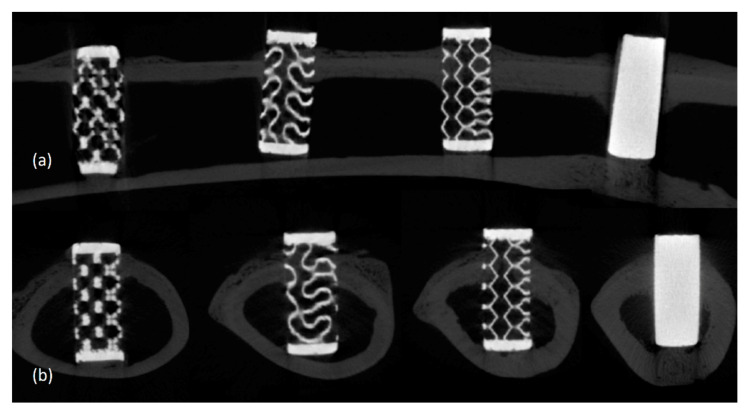
Micro-computed tomography findings of the titanium specimens with four different pore designs. (**a**) Longitudinal directional images (Dode, Gyroid, Octadens, and Solid (sham)). (**b**) Axial images (Dode, Gyroid, Octadens, and solid (sham)).

In all specimens of all groups, an uneven increase in radio-opacity in the pores was observed. This increase in opacity was lower than that of the cortical bone and higher than that of the marrow portion. There were no visual radiographic differences according to the time of sacrifice. No bony resorption pattern was observed around the specimens (Figure 4).

### 3.3. Histological Findings

**Figure 5:**
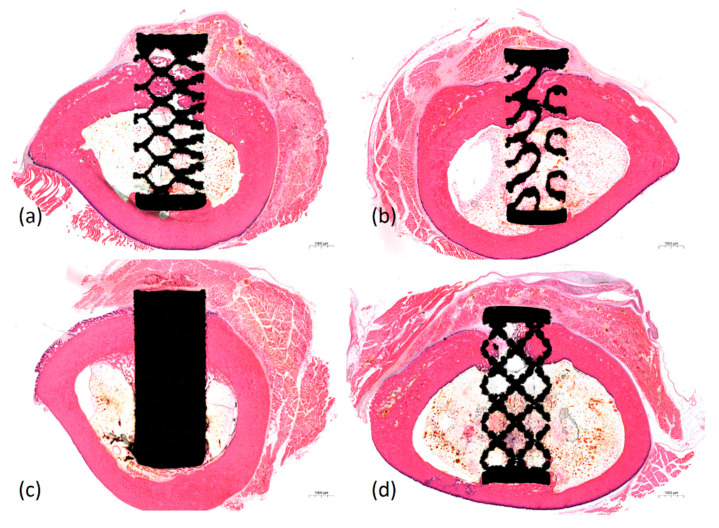
Histological images of the titanium specimens with four different pore designs. (**a**) Octadens pore design. (**b**) Gyroid pore design. (**c**) Solid. (**d**) Dode pore design (H&E staining, X1.0).

Histological findings obtained using H&E staining showed that inflammatory reactions associated with titanium specimens did not occur in any of the groups. Necrosis, infiltration of immune cells, and granuloma formation were not observed. Over time, more bony growth and maturation appeared in the pores. An interesting observation is that the compact bony tissue was penetrated into the pores adjacent to the cortical layer and spongious bony tissue was penetrated into the pores adjacent to the marrow layer (Figure 5).

### 3.4. Statistical Interpretation of Bone Conduction Capacity

Based on the difference in grayscale level, the percentage of new bone in the pore structure was observed to be approximately 25% (Table 1). There was no difference in the amount of newly formed bone according to pore design at 2, 4, and 6 weeks (*p* > 0.05). In addition, no difference in the amount of new bone formation was observed with increasing time within the same pore design for all three designs (*p* > 0.05).

The surface density of the new bone in the pore structure was observed to be approximately 8 mm^2^/mm^3^ (Table 2). There was no difference in the surface density of new bone according to pore design at 2, 4, and 6 weeks (*p* > 0.05). In addition, no difference in the surface density of new bone was observed with increasing time within the same pore design for all three designs (*p* > 0.05).

## 4. Discussion

As mentioned in the introduction, when titanium is applied as a porous structure, a space for bone formation and regrowth can be provided [5]. In addition, the porous structure lowers the weight of the substitute, lowers the stiffness to avoid stress shielding, has an elastic modulus similar to bone, and allows nutrient and oxygen exchange to occur along the connected pores [3,4,16].

However, until the introduction and development of 3D printers, imparting porosity to titanium has undergone many trials and errors. Casting and milling methods, which were the simplest manufacturing processes in the early days, are unsuitable for making homogeneous and fine pores in the internal structure of titanium [6]. To overcome such process limitations, various methods for manufacturing porous titanium have been studied, including hot isostatic pressing [17], metal injection molding [18], spark plasma sintering [19], space holder technique [20], plasma spraying [21], anodic dissolution [22] and powder metallurgy [23]. However, some techniques can produce only cavities and not interconnected pores. In addition, constructing a small and homogeneous pore structure using the above-mentioned methods was difficult.

3D printing, which is a revolutionary manufacturing method, has made it possible to overcome existing manufacturing imperfections. Unlike early printing methods, where only plastic materials were used, metal materials can now be processed through electron beam melting or SLM methods. In this study, the specimens were processed using SLM. The best advantages of constructing a bone substitute with titanium material having a porous structure using 3D printing are homogeneity and microstructure. An open and interconnected porous structure is typically needed for bone conduction with pore sizes ranging from 200 to 500 μm [24,25] and porosity ranging from 60% to 90% [26]. In addition, pore sizes larger than 300 μm could promote the formation of new bone and capillaries [27,28]. SLM allows for the production of bone implants made of metals such as, titanium and titanium-based alloys with a strut size as small as 200 μm [27]. Until now, residual powder, the so-called balling phenomenon, is a problem in the manufacturing of metal additives that causes adhering of particles in the 3D printing process. In other study, it has been reported that the use of hydrofluoric acid could overcome this problem [29].

The titanium specimens used herein were not subjected to special surface treatments. In fact, the current, widely used sandblasted or acid-etched techniques are known to increase the biocompatibility of titanium by maximizing the surface roughness of commercially pure titanium or titanium alloys [30]. Despite the advantages of such surface treatments, we did not perform it in consideration of the simplicity of the manufacturing process, and as excessive surface treatment may cause deformation of the set pore design. We envisioned that all used surfaces were treated the same to avoid additional effects of the surface treatment. Actually, according to one study, SLM-fabricated structures with native, sandblasted, acid-etched, polished, or vibratory grinded surfaces show identical cyto-compatibility with osteoblast compared with conventional titanium surfaces [27].

In this study, statistical differences in bone formation according to pore design were not identified. Regardless of the pore design, the percentage of new bone and surface density in the pore structure were observed in approximately 25% and 8 mm^2^/mm^3^, respectively, over 6 weeks. Research on titanium pore design has been actively conducted, even before the introduction of 3D printing. Under in vitro examination, there was a study that demonstrated better bone formation in square-shaped pores [11], and another study showed that tissue amplification was superior in hexagonal-shaped pores [31]. One study indicated that a larger pore throat size, which affects the interconnection, is more advantageous for bone ingrowth [32], and another study indicated that the straight edges and convexities in the pore design are the least favorable for cell growth [12].

The results were also inconsistent in a comparative study on the pore design of porous titanium made with 3D printing that can be searched in the current medline database. Deng et al. studied porous titanium manufactured by the SLM method with four pore designs (diamond, triangular, circular, cubic) having a size of 650 μm and a porosity of 65%. In a specimen of diamond pore shape, more bone formation was observed in animal experiments, and slower flow rates were observed with computational fluid dynamics, which would help blood vessel growth [33]. In addition, Zhao et al. compared the octahedral and tetrahedron structures of porous titanium fabricated by the SLM method and demonstrated that cell proliferation was better in the octahedral unit with immunofluorescence [34]. Van Bael et al. studied the pore shape and curvature, and their results showed that an obtuse angle between the pores was more prone to cause cell blockage than an acute angle [13].

Although statistical significance was not observed herein, several peculiarities were discovered. Histologically, ingrowth of the compact bone was observed in the specimen area close to the cortical layer, and ingrowth of the spongeous bone was observed in the specimen area close to the marrow layer. In addition, no difference in bone formation according to the implantation period was observed radiographically, suggesting that the maturation and calcification of bone tissue proceeded slowly before 6 weeks.

In this study, experiments related to cell affinity such as cell adhesion were not performed. Because except the pore design, the material and surface treatment were the same between the specimens, it was thought that there would be no significant difference in cell affinity. Even considering the existing literature, there is no disagreement about the high cell affinity of 3D-printed porous titanium. At 1 to 2 weeks after cell culture, more cell binding, filopodia, and lamellipodia were observed comparing to initial time [35]. In addition, it is reported that there was no significant difference in this tendency even when the specimen pore size was changed [25].

In addition to cell affinity, trabecular anisotropy may be considered in order for future porous specimens to adapt to loading conditions. As there are studies on bone mechanical integrity according to the open site of the pore [36,37], trabecular anisotropy according to the pore design should be investigated in the future. Besides that, the lack of sample size, short-term observation period, and non-loading environment are limitations of this study. Moreover, due to the absence of a device capable of superimposing the STL file used in the scaffold design and the CT data of the actually manufactured specimen, the printing accuracy could not be measured in this study. Studies addressing these aspects in the future would lead to more reliable results with regard to the effect of pore design on bone formation, which could not be observed herein.

## 5. Conclusions

High-porosity 3D printed titanium scaffolds that were constructed using the SLM method have high biocompatibility and bone conductivity in vivo. During the 6-week observation period, the proportion of new bones in the 3D-printed titanium scaffold was approximately 25%. However, radiologically and histologically, differences in bone formation according to the three types of pore design or implantation period were not observed.

## Figures and Tables

**Table 1:** New bone volume (%) of 3D-printed porous titanium with variable pore design.

	Octadense	Gyroid	Dode	*p*-Value
2 weeks	24.888 ± 0.872	25.069 ± 1.259	24.990 ± 2.715	0.957
4 weeks	27.874 ± 2.184	25.171 ± 1.656	26.527 ± 2.311	0.491
6 weeks	26.835 ± 2.078	27.591 ± 1.719	27.433 ± 4.143	0.733
*p*-value	0.252	0.193	0.670	

**Table 2:** Surface density of new bone (mm^2^/mm^3^) of 3D-printed porous titanium with variable pore design.

	Octadense	Gyroid	Dode	*p*-Value
2 weeks	8.073 ± 0.170	8.100 ± 0.055	8.278 ± 0.205	0.393
4 weeks	8.117 ± 0.399	7.727 ± 0.081	8.263 ± 0.127	0.099
6 weeks	7.765 ± 0.181	7.746 ± 0.341	7.989 ± 0.084	0.587
*p*-value	0.193	0.301	0.113	

## Data Availability

All results generated or analyzed during the present study are included in this published article. Data and materials will be made available upon request via email to the first author (davidjoy76@gmail.com).
